# Emerging Paradigms in Cholesteatoma: From a Traditional Approach to Personalized Therapy

**DOI:** 10.3390/ijms26199545

**Published:** 2025-09-30

**Authors:** Adina Zamfir-Chiru-Anton, Dana Manda, Dan-Cristian Gheorghe

**Affiliations:** 1 “Grigore Alexandrescu” Emergency Hospital for Children, 011743 Bucharest, Romania; zamfiradina@yahoo.com; 2 Faculty of Medicine, “Carol Davila” University of Medicine and Pharmacy, 050474 Bucharest, Romania; dan.gheorghe@umfcd.ro; 3 “C.I. Parhon” National Institute of Endocrinology, 011863 Bucharest, Romania; 4 “Marie Curie” Emergency Hospital for Children, 077120 Bucharest, Romania

**Keywords:** cholesteatoma, personalized medicine, middle ear

## Abstract

Cholesteatoma is a prevalent disease affecting both children and adults. In this review, we present the recent findings related to the molecular mechanisms involved in cholesteatoma and discuss how researchers can target new molecules to treat this disease. These new approaches illustrate the paradigm shift from a primarily surgical solution to a biological “control and prevent” strategy.

## 1. Introduction

Cholesteatoma is a prevalent disease affecting both children and adults, which includes the formation of an abnormal epithelial pouch in the middle ear and mastoid cavities, with accumulating keratin debris and lack of its clearance [1]. The incidence of this condition varies, according to different studies; however, it affects approximately 0.09% of the global population [2]. Cholesteatoma typically develops as a chronic disease and can have significant complications. For example, otogenic brain abscesses secondary to middle ear cholesteatoma have a mortality rate of 8–26.3% [3]. From a clinical perspective, the disease remains asymptomatic until its late stages of development. Visual screening of the tympanic membrane serves as a highly effective preventive measure, facilitating the identification and treatment of cholesteatoma. Determination is paramount when cholesteatoma is suspected inside the middle ear and mastoid cavities. Unfortunately, only imaging can show the signs of the disease. Bony erosion and clearly limited lesions inside the temporal bone cavities indicate the consequences of the pathological process [4]. For screening purposes, no other laboratory investigation can provide a useful association with middle ear cholesteatoma. Even the newly accepted methods of disease monitoring after surgery (e.g., Magnetic Resonance Imaging (MRI)) cannot avoid false positive and false negative results when diagnosing cholesteatoma recurrences [5]. Management involves surgical removal of the epithelium from all the tympanic and mastoid cavities, along with the repair of the eardrum and the ossicular chain erosions caused by the cholesteatoma lesions. Despite its benign histological appearance, cholesteatoma exhibits an aggressive and invasive behavior characterized by hyperproliferation, increased migratory capacity, abnormal differentiation of keratinocytes, and bone erosions.

This paper aims to review the current management and molecular mechanism of cholesteatoma. Understanding the complex molecular interactions may lead to new reliable biomarkers and molecules that can target the altered mechanisms of this disease.

## 2. Pathogenesis, Classification, and Treatment

The pathogenesis of cholesteatoma is still unknown, although the condition has been known for some time [6]. To better the delineate possible mechanisms involved in keratinocyte hyperproliferation, migration, and destruction of adjacent tissues, more study should be undertaken of the anatomical and clinical forms of the disease.

**Figure 1:**
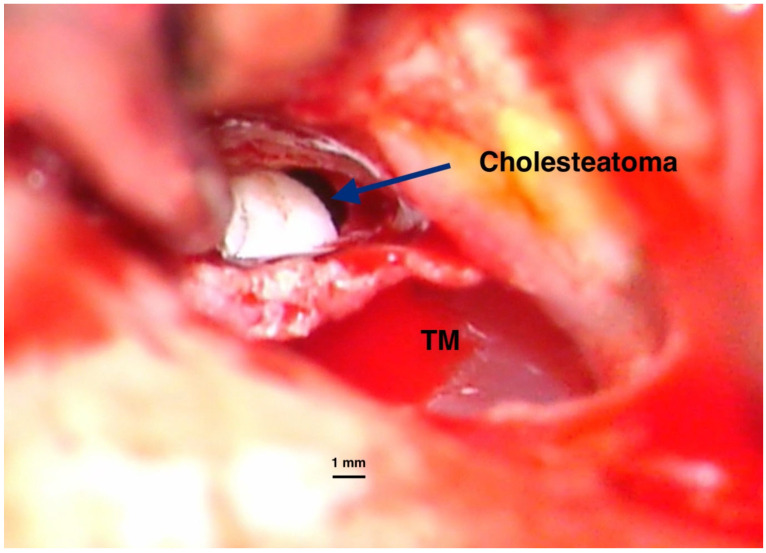
Intraoperative imaging of a congenital cholesteatoma of the right ear. Tympanic membrane and anterior part of the external canal wall skin have been basculated posteriorly to expose the lesion. No perforation of the eardrum was present before surgery. Congenital cholesteatoma (TM—Tympanic membrane). Picture taken with Zeiss TIVATO 700 microscope (ZEISS Microscopy, Jena, Germany). (from D.C.G’s. personal collection).

**Figure 2:**
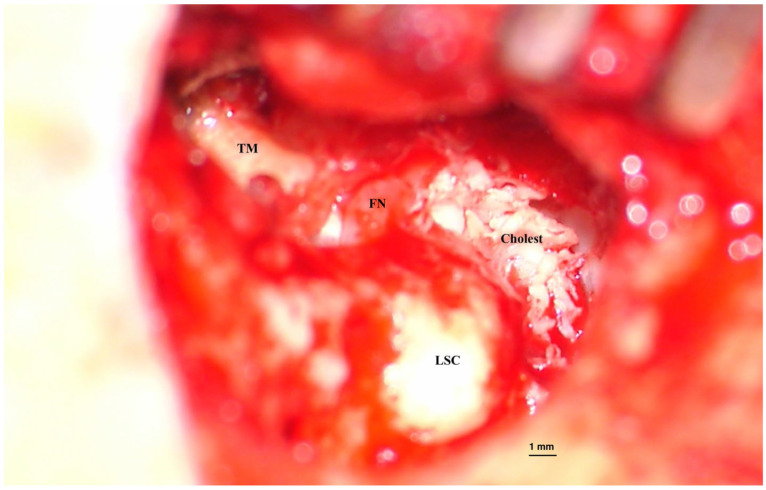
Acquired cholesteatoma. Intraoperative image of surgery for an acquired cholesteatoma on the left ear. Epithelial matrix covered with keratin debris is visible on the inner wall of the middle ear and mastoid cavities. Bone erosions can be observed at the periphery of the lesion, suggesting the mechanism of invasion inside tympanomastoid spaces. (TM—Tympanic membrane, FN—facial nerve, LSC—Lateral semicircular canal). Picture taken with Zeiss TIVATO 700 microscope (ZEISS Microscopy, Jena, Germany). (from D.C.G’s. personal collection).

Classically, cholesteatomas are classified into three types [1]:

- Congenital and rare, with an intact tympanic membrane (Figure 1);
- Primary acquired, developed from a retraction pocket, due to Eustachian tube dysfunction or chronic otitis media (Figure 2);
- Secondary acquired, with tympanic membrane perforation or trauma; this is not necessarily a typical cholesteatoma lesion, and some authors use the term middle ear (or mallear) epidermosis to describe it [7].

The difficulty in cholesteatoma classification arises from the different theories proposed to explain the disease occurrence and progression. No theory fully covers all the clinically and surgically encountered situations at the level of the middle ear and mastoid cavities. From a pathological point of view, the integrity of the eardrum determines the disease management and prognosis. Complete separation of the epithelial lesion from the tympanic and external canal wall skin in congenital cholesteatoma can only be explained by the inclusion hypothesis, while in the acquired form with bone erosion, the disease progression can be linked to local chronic inflammatory processes and epidermal hyperproliferation. Modern studies focus on this acquired form of the disease, since its management and evolution are burdened by high rates of residual and recurrent disease [8]. Some cholesteatoma-associated perforations of the eardrum are the consequence of long-term unrecognized disease with spontaneous drainage or of tympanotomy performed for misinterpreted otoscopic images, in which cases, the classification as congenital or acquired lesions becomes difficult.

Cholesteatoma management is always surgical. Medical treatment has been used as adjuvant in cases with infectious lesions of the external auditory canal. Due to the chronic evolution of cholesteatoma, emergency interventions are rare and include only complicated cases, such as facial nerve damage and intracranial infections [9]. Most other cases need a planned approach for the complete removal of the epithelial lesions in the tympano–mastoid cavities and the best possible reconstruction of the tympanic membrane and of the ossicular chain.

Post-surgical long-term follow-up is compulsory in cholesteatoma of the middle ear and mastoid. Different rates of residual lesions have been described after cholesteatoma removal, from 7.6% to 25% [10,11]. Recurrences are also possible, at a rate of 2.3% to 17%, depending on the surgical technique used for management [10,12]. Some authors have recorded similar recurrence rates regardless of the surgical technique [13,14]. Different studies have reported varying data on this topic (see Table 1).

From Table 1 we can say that studies reporting the recurrence rate in cholesteatoma showed that, regardless of the technique used, in children the recurrence rate is greater than 20%, which means that one in 5 children will have a recurrence, which is not negligible.

**Figure 3:**
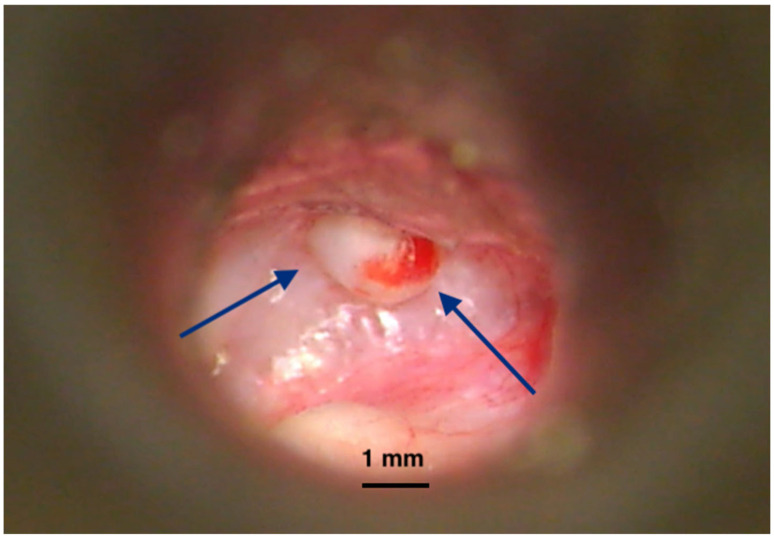
Recurrence of cholesteatoma in an open mastoid cavity. Postoperative imaging of an open mastoidectomy cavity after cholesteatoma surgery. The lining of the cavity is healed but at a certain location we notice a cholesteatoma recurrence (pearl-like) (blue arrows) developing and covered by a normal epithelium. Left untreated, it will create bone erosion underneath and enlarge, destructing the remaining of the temporal bone. Picture taken with Zeiss TIVATO 700 microscope (ZEISS Microscopy, Jena, Germany). (from D.C.G’s. personal collection).

The time between surgical removal and disease recurrence can vary greatly; hence, continuous monitoring over the long term is required [16,17]. Since only clinical examinations and imaging (MRI) are useful in this regard, medical resources and financial burden are critical for this disease. The statistics report a more aggressive disease in children [14]. In addition, due to their longer life span, more active diagnosis and management protocols are required. There have been many publications reporting the risk factors for local recurrence. Different locations of the disease within the temporal bone have been reported with varying rates of residual/recurrent lesions (Figure 3), leading to an extensive classification and staging of the cholesteatoma [18]. Surgery of the middle ear (e.g., ventilating tubes, cochlear implants) could also explain the genesis of a small number of cholesteatoma cases inside the tympanic and mastoid cavities [19,20,21].

The surgical management of cholesteatoma varies, with some favoring limited and more conservative operative techniques. Lower recurrence rates in open techniques provide a longer disease-free time in patients. However, the data in Table 1 show that even with open cavity mastoidectomy (CWD) surgery, there are still significant rates of recurrences, and no anatomical explanations can account for this situation. This justifies ongoing investigations into this disease for future management options.

Several theories have been proposed for the formation of acquired cholesteatoma. However, not all these theories provide sufficient evidence for verification. Some of the proposed theories, as outlined in Table 2, have been substantiated by published data and have been extensively discussed over time.

Metaplasia theory presumes that the middle ear mucosa suffers a squamous epithelium transformation due to local chronic inflammation. However, gathered data prove the origin of cholesteatoma epithelium to be the external canal skin [28]. Hyperplasia theory promotes the idea of epithelial proliferation secondary to inflammatory changes in the middle ear spaces, which is supported by the lesions occurring with an intact tympanic membrane [29]. Retraction pocket or invagination theory is the most widely accepted and assumes that middle ear conditions, such as the negative pressure induced by Eustachian tube dysfunction, determine alterations in the tympanic membrane anatomical position and structure [24]. Some authors have proposed a new theory that involves traction of the epithelium from the eardrum by the opposite middle ear mucosa [26]. It is also possible to have cholesteatoma lesions occurring after posttraumatic injury by means of epithelial implantation into the middle ear spaces; for example, fractures of the temporal bone can be accompanied by epithelial implantation in the middle ear space through a perforated eardrum [30]. The same condition can occur after tympanic membrane (e.g., ventilation tube insertion) or other mastoid surgery.

## 3. Molecular Mechanisms Involved in Cholesteatoma

In acquired cholesteatoma, the pathophysiology and molecular mechanisms involve a complex interplay of chronic inflammation [31,32], epithelial hyperproliferation [33], extracellular matrix (ECM) remodeling [30], and bone erosion [34]. Recent research has significantly advanced our understanding of its molecular and cellular basis, including dysregulated keratinocyte proliferation and chronic inflammation, matrix metalloproteinase increased activity, oxidative stress [35], osteoclastogenesis [36], and epigenetic modulation including miRNAs [37] (Table 3).

In this section we present the molecular mechanisms involved in cholesteatoma and in the next section we will present the potential applications of these findings for molecular diagnosis and treatment of the disease.

### 3.1. Inflammation

Inflammatory cytokines are significantly upregulated in cholesteatoma tissues, compared with normal skin or middle ear tissues [37]. Shiwa (1995) suggested that elevated IL-1alpha levels might be corelated with the granulation tissue surrounding the cholesteatoma matrix and might stimulate epithelial cells’ hyperproliferation [38]. Similar findings were reported by Schilling in 1992 [39]. Conversely, Serban (2021) reported lower serum levels of IL-1alpha in cholesteatoma cases [40]. A subsequent study by Dambergs (2024) demonstrated elevated levels of IL-1alpha in cholesteatoma tissues relative to normal skin [41]. It appears that IL-1 is produced in epithelial cells in response to stimulation from the subepithelial layers [42].

Other pro-inflammatory cytokines, such as IL-1, IL-6, and IL-8, have been identified in both the epithelial and stromal layers of cholesteatoma and have been significantly correlated with granulation tissue and bone destruction. A possible protective role has been hypothesized for transforming growth factors [43]. Liu (2014) observed higher levels of IL-6 in cholesteatoma epithelium but could not establish a link to the adjacent bone destruction and supported only a hyperproliferative effect in cholesteatoma [44]. Leal Alves (2004) suggested that high concentrations of IL-6 induce osteoclast formation, and IL-8 is a promoter for production of matrix metalloproteinases [45]. These interleukins may induce fibroblast activation, leading to the increased secretion of other cytokines such as epidermal growth factor (EGF), tumor necrosis factor-alpha (TNFα), and tumor growth factor-alpha (TGFα) [45]. Other cytokines, such as IL-17, have been recorded in higher concentrations in cholesteatoma lesions due to the presence of CD4-positive lymphocytes showing local bone destructions [46].

Inflammatory cytokines and mediators produced by the immune system trigger osteoclast activation and tissue remodeling, which are key features of cholesteatoma progression. However, not all mediators contribute to cholesteatoma destruction. One study investigated the role of Toll-like receptors 4 (TLR4) and showed they promoted local inflammation and bone destruction via RANKL [47]. Other authors have proposed that transforming growth factors beta 1 and 2 may exert a protective effect, underscoring the intricate molecular interactions implicated in the local inflammatory response within cholesteatoma [43].

Heat shock proteins (HSP) respond to cellular stress and inflammation in cholesteatoma, which involves cytokines such as IL-1β, IL-6, IL-8, and TNF-α and growth factors such as EGF, KGF, TGF-α/β, and VEGF. These molecules modulate keratinocyte proliferation, differentiation, angiogenesis, and bone resorption, processes in which HSPs participate by stabilizing proteins and mediating stress responses [48].

Small sample sizes, the lack of prospective biomarker-driven outcome studies, and the absence of data on the impact of surgery or adjuvant therapies limit the power of these results (Table 4).

No clinical studies exploring the anti-inflammatory drugs in cholesteatoma exist till now. In vitro use of TLR4 inhibitors (Lipopolysaccharide, LPS, from *R. sphaeroides*) showed a significant decrease in the inflammatory response in culture cells but its use in a clinical setting needs to be investigated [47].

### 3.2. Matrix Degradation

Matrix metalloproteinases are enzymes responsible for the degradation of the extracellular matrix, thereby contributing to tissue damage in cholesteatoma [31]. This process facilitates the local invasion and destruction of the surrounding tissues. Elevated levels of MMP-2 have been documented in cholesteatoma tissues, prompting various hypotheses regarding the disease’s pathogenesis [49]. Some studies, such that of Rocha Morales (2007), indicate that MMP-2 protein expression is higher in aggressive forms of cholesteatoma [50]. Conversely, Kan (2021) observed increased MMP-2 mRNA expression in advanced-stage cholesteatomas in fibroblasts and local inflammatory cells but found no association with residual or recurrent disease [51]. Additional studies have measured the serum levels of MMP-2 and MMP-9, correlating their levels with ossicular chain erosion in cholesteatoma [52]. Further, the significantly elevated tissue expression of MMP-9 correlated with cholesteatoma recurrence [20]. Lei (2023) reported markedly higher MMP-14 levels in cholesteatoma specimens compared with normal skin, correlating the recorded data with the extent of bone destruction [53]. Conversely, Mehta (2007) found no significant increase in the MMP-8 and MMP-13 levels in cholesteatoma epithelium relative to the normal external ear skin [54].

MMP-2 and MMP-9 cleave the denaturated collagen (gelatin) and other proteins from the extracellular matrix. MMP-8 and MMP-13 cleave fibrillar collagen types I–IV. MMP-14 is a membrane matrix metalloproteinase that can activate MMP-2 and MMP-9 [55]. As the cholesteatoma core is skin and keratin surrounded by inflammatory fibrous tissue containing collagen and fibroblasts, we may suppose that there is more degraded than structured collagen. Due to their complex interactions, it is challenging to determine the role of each MMP because they act synergistically with other proteins. Further research is needed to explain the complex interrelations between inflammation molecules and MMPs to target the abnormal matrix degradation with efficient and specific pharmacological MMP inhibitors. A summary of gathered data is presented in Table 5.

There are inconsistencies about matrix metalloproteinases roles in cholesteatoma, since some studies investigated tissue concentrations, others serum levels. Serum markers can be utilized to monitor disease recurrence, whereas obtaining tissue samples requires surgical intervention and may obscure medical management. Also, no clear cause effect can be established with regard to MMPs in relation to the inflammatory processes from cholesteatoma lesions.

### 3.3. Osteoclast Activation

This hypothesis has been proposed by authors demonstrating histological evidence of osteoclasts in large numbers in cholesteatoma lesions [56]. In the presence of local inflammatory cytokines, the upper regulation of RANKL in perimatrix fibroblasts was recorded. Along with RANKL, other proteins involved in calcium homeostasis such as PTHrP were significantly found at increased levels compared with normal postauricular skin [57]. PTHrP promotes macrophage differentiation into osteoclasts, induced by RANKL, promoting bone destruction in cholesteatoma [58]. The correlation between the expression of these proteins and bone-destructive lesions is therefore unequivocally established. RANKL is expressed within the fibroblasts located in the vicinity of the matrix [58]. Some published data suggest that other local mediators, such as Activin A, are needed to activate synergistically with RANKL to induce osteoclast formation [36]. In addition, a deficient transcription factor AP-2-beta (TFAP2B) could be involved in osteoclast differentiation with resulting bone destruction [59]. The importance of such reported data cannot be overemphasized since potential drugs could target both the mediators (e.g., follistatin against Activin A) and the fibroblasts producing them.

### 3.4. miRNA Dysregulation

As microRNAs regulate gene expression at the post-transcriptional level, many researchers have turned their attention to the miRNA expression profile in cholesteatoma. Multiple miRNAs have been investigated. Gong described an upregulation of RANKL in fibroblasts via miRNA-17, and this effect, in turn, promotes osteoclast differentiation [60]. The same hypothesis was supported by Imai, who found that mRNA stimulated the upload of RANKL into the fibroblasts from the cholesteatoma matrix. He also recorded the presence of more osteoclasts near cholesteatoma lesions, compared with the control bone [56]. One of the first miRNAs significantly highly expressed and observed in cholesteatomas was miR-21, which negatively regulates the expression of the phosphatase and tensin homolog PTEN (a protein that inhibits cell growth) and PDCD4, a protein involved in programmed cell death [61,62]. Any factor that contributes to miR-21 inhibition, such as STAT3 inhibitors, can modify its activity and inhibit the proliferative activity of the epithelial layers of cholesteatoma.

miR-802 is another microRNA upregulated under inflammatory conditions (e.g., TNF-α, IL-6, and RANKL stimulation). It suppresses PTEN and promotes keratinocyte proliferation through protein kinase B (Akt) signaling [63].

On the other hand, miR-142-5p was recorded as downregulated in cholesteatoma. Its normal role is to inhibit cyclin-dependent kinase 5 (CDK5), a kinase involved in inflammatory cytokine release. Its suppression amplifies local inflammation by increasing IL-6 and TNF-α production [64]. These miRNAs form a feedback loop in which inflammatory signals alter miRNA expression, and miRNAs in turn modulate the inflammatory response and cell behavior.

miRNAs that inhibit epithelial hyperplasia exist and are suppressed in cholesteatoma, such as miR-203a, a keratinocyte-specific miRNA, which is downregulated in cholesteatoma. This leads to the excessive proliferation of keratinocytes and decreased apoptosis [65].

Let-7a is a micro-RNA that plays a tumor-suppressive role by inducing G1 cell cycle arrest and apoptosis. It also suppresses the invasion and migration of keratinocytes. It is negatively correlated with miR-21, suggesting a regulatory balance between proliferation and differentiation [66]. miR-21 has been found highly expressed in cholesteatomas compared with normal skin [42].

Another micro-RNA (miR-10a-5p) negatively regulates *PIK3CA.* Phosphatidylinositol-4,5-bisphosphonate 3-kinase catalytic subunit α (*PIK3CA*) is a gene that encodes the p110α protein, a catalytic subunit of the PI3K enzyme that is involved in cell growth, proliferation, and survival [67].

Downregulation of miR-34a and miR-125b abrogates intrinsic apoptotic priming via BCL-2, survivin, and STAT3 upregulation [68]. In addition, the latest studies propose that miR-34a targets epidermal growth factor receptors (EGFR) to achieve its antiproliferative effect [69].

A recent study identified 121 significantly differentially expressed miRNAs in cholesteatoma compared with normal tissues. Here, 56 miRNAs were upregulated, and 65 were downregulated. The study identified *TGFBR2, MBNL1*, and *NFAT5* as potential key target genes in middle ear cholesteatoma [70].

Due to so many differentially expressed miRNAs in cholesteatomas, it is difficult to precisely delineate their role in cell cycles, apoptosis, and cell differentiation. More investigative efforts are needed before clinical trials can propose medical agents to alter the disease course or prevent it (Table 6).

### 3.5. Oxidative Stress

It has been suggested that other local factors that influence molecular dynamics could play a role in the development of cholesteatoma. Oxidative stress can be increased, and antioxidant enzymes can be decreased in cholesteatoma patients [71]. The same conclusions were presented by Garça as well, although they were not statistically significant [72]. An important finding from the same group showed that oxidative stress, evaluated in the circulation (serum), was significantly higher in patients with cholesteatoma [73]. Later investigations into the roles of vitamin A and E did not support associations between their lower levels and the risk for cholesteatoma [74].

Hypoxia is considered one of the triggers for cholesteatoma progression [75]. Hypoxia-inducible factor 1 (HIF1A) is present in high concentrations in the basal layer of keratinocytes from the cholesteatoma matrix. Some authors have associated higher levels of this mediator with the likelihood of relapse after surgery [75]. It is obvious from the pathology of lesions that the cholesteatoma matrix and its surrounding tissues are compressed in a tight space predisposing them to hypoxia, especially if keratin debris accumulates, and its drainage is impaired. Along with HIF1A, upregulation of Endothelin Converting Enzyme 1 (ECE1), an enzyme that is involved in the synthesis of endothelin and other vasoactive peptides, was shown, demonstrating a complex chain of events that can be triggered or maintained by hypoxia and its inducing factors [76].

PPARβ/δ is a key regulator promoting cholesteatoma keratinocyte proliferation through the PDK1/PTEN/AKT/GSK3β/Cyclin D1 pathway, while PPARγ is upregulated and may influence differentiation and proliferation. ROS contributes to cholesteatoma pathology likely through oxidative stress and inflammation, with possible but not fully defined crosstalk with PPAR signaling pathways [77]. SIRT proteins could theoretically modulate p53-mediated pathways, but direct studies on SIRT/p53 interaction in cholesteatoma are lacking. The Nrf2/Keap1 signaling pathway is a key cellular defense mechanism against oxidative stress, with Nrf2 being a transcription factor that regulates antioxidant responses and Keap1 acting as its negative regulator [78]. While specific studies on Nrf2/Keap1 in cholesteatoma are limited, the pathway is generally recognized for its role in protecting cells from oxidative damage and inflammation, which are processes involved in cholesteatoma’s pathogenesis [79].

Oxidative stress and inflammation converge in multiple proliferative pathways through both direct mechanisms (e.g., oxidative damage) and indirect mechanisms (cytokine and growth-factor mediated signaling). The analyzed studies, although heterogeneous and limited by their small sample size, reported that the identified molecules are mediators of the interplay between oxidative stress inflammation and tissue remodeling. Further integrative and translational research is needed to optimize intervention strategies.

### 3.6. Epigenetic Alterations

Maybe the most important anatomic alteration seen in middle ear cholesteatoma is abnormal epithelial behavior, leading to destruction of the ossicular chain and the temporal bone structures. Even with proper ventilation of the skin lining with open cavities after surgical management, some recurrences cannot be explained, except by the disrupted homeostasis of the epithelial cells [80]. Cellular receptors could be involved in epithelial cell proliferation. Keratinocyte growth factor (KGF) has been associated with the recurrence of cholesteatoma in 80% of cases [81]. It seems this step could be linked to histone modifications and FOXC2 expression. The involvement of KGF’s increased expression in cholesteatoma was also demonstrated by Harabagiu et al. in a study that showed that paracrine interaction between KGF and its receptor increased the aggressiveness of the disease [82].

Histone modifications and RNA methylation have been investigated and demonstrated to affect gene expression and epithelial cell behavior. Histone H3 trimethylation at lysine 4 (H3K4me3) is highly expressed in cholesteatoma tissues and can be influenced by the menin-mixed lineage leukemia 1 (MLL1) inhibitor [83]. Although this evidence was based on animal studies, the potential for disease prevention after surgical removal was introduced.

The same author demonstrated that H3K27 (histone H3 at lysine 27) acetylation is a super-enhancer activity for the *FOXC2* (forkhead box C2) locus, driving FOXC2 upregulation in cholesteatoma samples [84]. FOXC2 is involved in and has been proposed as an independent prognostic factor in gastro-esophageal and lung cancers [85,86]. It has also been associated with angiogenesis [87]. The multiple implications of the FOXC2 in different cellular processes makes it difficult to understand its detailed role in the mechanisms of cholesteatoma growth; however, its cellular properties could sustain increased epithelial proliferation, under the signaling of local molecules such as KGF.

### 3.7. Immune Cell Infiltration

Cholesteatoma research on the receptors present in immune cells has been conducted due to the evidence suggesting the presence of elevated local inflammatory mediators compared with normal skin. Triggering receptors expressed on myeloid cells (TREM) were tested in acquired cholesteatoma lesions and seemed to be integrated in a chain of events including IL-1β, IL-6, MMP-2, and MMP-8, finally concluding with osteoclast activation [88].

Invasive cholesteatomas showed immune cell infiltration, mainly with CD3+ cells (T lymphocytes) and CD68+ (monocytes and macrophages) but not CD20 or CD1a(+), suggesting a cell-mediated immune process [89].

The proliferative process of the cholesteatoma matrix is enhanced by local mast cell infiltration (CD117). These cells stimulate angiogenesis and contribute to increased epithelial survival. An association was also demonstrated between their higher numbers and bone erosions in cholesteatoma [90].

Some authors have considered the immune response as a target since cell mediated inflammatory processes have been demonstrated in cholesteatoma lesions. Still, tacrolimus use in lab experiments failed to obtain an anti-inflammatory action, leaving room for other hypothesis in the pathogenesis of this disease [91].

### 3.8. Apoptosis

Studies that compared cholesteatoma tissue with normal external ear canal skin showed that cholesteatoma exhibits an increased rate of both keratinocyte proliferation and apoptotic cell death compared with normal skin. This balance is important since despite hyperproliferation, cholesteatoma cells retain their ability to undergo programmed cell death [92]. Certain microRNAs (e.g., miR-508-3p) regulate cholesteatoma cell proliferation and apoptosis by targeting key signaling pathways such as PTEN/PI3K/AKT, emphasizing the complex molecular regulation of cell death processes [93]. p53 and p21 are apoptosis- and cell cycle-related proteins, and their expression levels have been investigated in congenital and acquired cholesteatomas [94]. Upregulation of p21 is noted in congenital cholesteatoma and may play a significant role in its development [94]. These markers reflect some pathophysiological mechanisms (apoptosis and cell cycle regulation) involved in cholesteatoma but have not been validated as standalone diagnostic biomarkers due to their variability and overlap with normal and other pathological processes.

## 4. Discussion and Future Directions

Despite advances in surgical techniques, there remain gaps in our understanding of cholesteatoma’s pathogenesis. It is also important to understand the postoperative disease, determining the mechanisms involved. Residual cholesteatoma means one or more remnant lesions are left in place during the surgical procedure, while recurrent disease implies reformation of the epithelial pocket with the accumulation of keratin debris. Young age and extensive disease at the time of the first surgery have higher chances of recurrence [14]. As yet, no correlation has been recorded regarding the surgical technique utilized in cholesteatoma management (canal wall up or down) and the result. Further, traditional models have failed to explain the clinical variability.

New theories about the pathogenesis of cholesteatoma hypothesize key molecular pathways: pro-inflammatory, osteolytic, epigenetic, stress response, and modulation of apoptosis. These pathways could explain the variable aggressiveness and recurrence rates and may provide targets for medical therapies instead of surgical procedures. They could be useful especially for preventing disease recurrence, as a good adjunct to surgical treatment.

The effort to elucidate the pathogenic mechanisms in cholesteatoma aims to identify sensitive and specific biomarkers that will be useful in the diagnosis, prognosis, and evaluation of the treatment efficacy.

Moreover, some molecules may be suitable as pharmacological targets.

However, the studies conducted to date have some weaknesses: the small number of samples analyzed and the heterogeneity of the evaluation criteria. In addition, there are very few studies that have followed the variation in potential biomarkers both in tissue and in serum. This approach is important for identifying the most reliable serum biomarkers, an essential step towards precision medicine and personalized therapy.

The macrophage differentiation into osteoclasts induced by PTHrP/RANKL, the complex and interrelated miRNA network, the immune cell infiltration, the epigenetic alterations, and the stress response represent findings that are changing the paradigm: we are viewing cholesteatoma as an inflammatory and proliferative process rather than simple epithelial cell accumulation. Using antagonists for TRL4 before surgical management could lower the bone erosions in cholesteatoma and lessen the operative burden [52].

Advances in the molecular profiling of tumors have revealed differentially expressed genes, specific miRNAs expression, epigenetic modifications, regulatory mechanisms, and a hypoxic stress response in cholesteatoma compared with normal skin tissue. These findings could help to determine therapeutic targets.

Inflammatory cytokines inhibitors can act by blocking cytokine receptors, inhibiting enzymes involved in the formation of active cytokines or capturing the cytokines by monoclonal antibodies. The therapies have been already approved as biological therapies in systemic inflammatory diseases.

Functional inactivation of TLR4 might effectively block pro-inflammatory signaling in cholesteatoma cells. There are TLR4 antagonists in clinical and preclinical studies [95]. The challenge for all therapies is to deliver them to target tissues.

While several potential HSP inhibitors have been identified there are not systematic studies to validate these molecules as therapeutic agents [96]. 

MMPs can be used as prognostic or recurrence markers. The MMP inhibitors have significant toxicity therefore further pharmacological research is needed.

Anti miR-17 treatment has proven the ability to reduce tumor cell proliferation and induce apoptosis in cancer [97]. There are preclinical tests of anti-miR-17 oligonucleotide for the treatment of polycystic kidney disease [98].

Inhibition of miR-21 function can be performed using site-selective artificial ribonucleases [99]. This can be applied to specifically inhibit all miRNAs that are upregulated in cholesteatoma as miR-802, miR-142-5p, For downregulated miRNAs nanoparticles delivery was tested for miR-34a on cell cultures [69].

H3K4me3 is an epigenetic marker found higher expressed in cholesteatoma. The therapeutic strategy was to inhibit MLL1 which is involved in H3K4me3 expression [83].

Some new technologies could be useful in personalized cholesteatoma management:

- Nanoparticle delivery systems for targeted anti-inflammatory treatment [69]. Nanoparticles allow localized drug delivery in the middle ear. Silver nanoparticles [100] or ZnO nanoparticles are widely used or their antibacterial properties. But ZnO nanoparticles proved to be efficient in drug delivery due to their low toxicity, biocompatibility and controlled drug release properties [101].
- RNA therapy to silence the key genes involved in pathogenesis [102]. These therapies silence disease-causing genes using small interfering RNAs (siRNAs) [103] or antisense oligonucleotides (ASOs) to block mRNA translation [104].
- Biodegradable matrices that can be applied during minimally invasive procedures to reduce inflammation and infectious risk. These can be used over exposed bone following cholesteatoma excision [105].

Although substantial gaps remain, the accumulating multi-omic evidence sets the stage for miRNA-driven diagnostics and combinatorial therapies that could one day complement canal-wall-down tympanoplasty and curb the high recurrence burden of cholesteatoma.

Topical 5-fluorouracil has shown promise in randomized controlled trials and demonstrated a significant reduction in tumor size [106]. Anti IL-6 agents are in Phase I clinical trials, showing reduction in inflammatory markers, though with some side effects [107].

There are already approved drugs that can target molecules involved in cholesteatoma occurrence [108], but clinical trials are needed to confirm their beneficial effects in this pathology.

**Figure 4:**
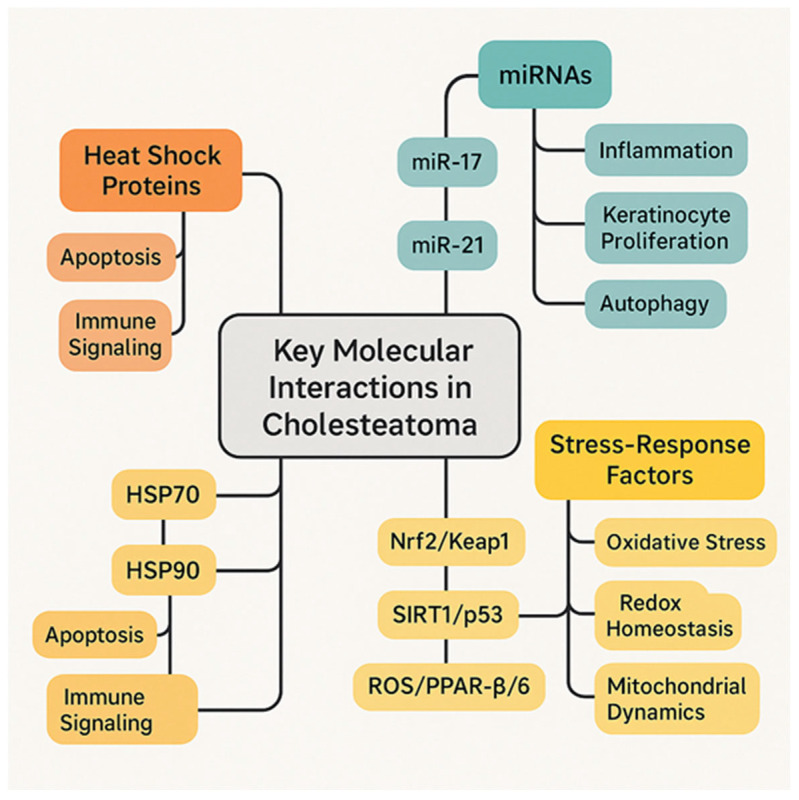
Key molecular interactions in cholesteatoma.

Despite these advances, research gaps remain to be addressed: first, biomarkers, especially serum markers that could identify patients at risk for aggressive disease or recurrence; second, regenerative approaches using engineered tissue; third, more therapeutic and innovative molecules in clinical trials (Figure 4).

All these new approaches illustrate the paradigm shift from a primarily surgical solution to a biological “control and prevent” strategy.

Searching for alternative methods to treat cholesteatoma involves several key aspects with a social impact:

1. Improving access and equity: Alternative treatments can help address social determinants such as the costs and addressability. This is important because these factors can influence access to conventional care, delays in diagnosis, and treatment outcome, especially for underserved populations.
2. Public health and awareness: Advancing non-surgical therapies increases the awareness of the community and healthcare providers, which can improve early diagnosis protocols, thus avoiding complications and longer treatments.

Overall, new innovative treatments for cholesteatoma can improve health equity, reduce the psychological and economic burdens, and enhance the quality of life through more accessible, less invasive, and effective treatment options.

To achieve this goal, it is necessary to concentrate and strengthen research efforts, which could be achieved by creating an international consortium.

## 5. Conclusions

Exploring comparative tumor biology, leveraging systemic immune modulation, and refining local-targeted therapies may converge to rewrite the clinical management of this disease. Preventing recurrence or the development of residual lesions can make a difference in the medical wellbeing of patients with cholesteatoma of the middle ear. Future investigations should prioritize uncovering pivotal molecular drivers, developing tools for early molecular diagnosis, and rigorously assessing the safety and efficacy of next-generation therapeutics in preclinical and clinical settings.

In this way, we can move from the traditional paradigm—surgical management—to an emerging paradigm based on molecular findings, through preventive and personalized therapies.

## Figures and Tables

**Table 1:** Rates of recurrence in pediatric cholesteatoma (more than five-year follow-up).

Study, Year	CWU (%)	CWD (%)	Unspecified (%)	No. of Patients
Piras, 2021 [10]	22.9	2.3		236
James, 2024 [14]	23.0	23.2		408
Møller, 2020 [15]			37.0	300
Stangerup, 1998 [16]			24.0	114
Kuo, 2012 [17]			19–25	146

CWU: canal-wall-up surgical technique; CWD: canal-wall-down surgical technique.

**Table 2:** Theories of the pathogenesis of acquired cholesteatoma.

Theory	Author (Year)
Metaplasia	von Tröltsch (1864) [22] and Wendt (1873) [23]
Hyperplasia	Lange (1925) [24]
Invagination (retraction pocket)	Wittmaack (1933) [25]
Implantation	Wullstein (1980) [26]
Mucosal traction	Jackler (2015) [27]

**Table 3:** Molecular mechanisms identified in cholesteatoma pathophysiology.

Mechanism	Description	Key Molecules
Inflammation	Chronic cytokine activity	IL-6, TNF-α, IL-1β, IL-8, HSP27
Matrix Degradation	Destruction of bone matrix and ECM	MMP-2, MMP-9
Osteoclast Activation	Bone resorption	RANKL, OPG, IL-17, PTHrP
miRNA Dysregulation	Alteration in epithelial gene expression	miR-21, miR-203
Oxidative Stress	Inflammation and cell migration	ROS, HIF-1α, PPARβ
Epigenetic Alterations	Histone modification and gene silencing	HDACs, miRNAs
Immune Cell Infiltration	Inflammatory signaling	CD3+, CD68+ cells
Apoptosis	Phosphorylated HSPs inhibition of apoptosis pathways	HSP60, HSP70, HSP27, p53, p21

IL-6: interleukin 6, TNF-α: tumor necrosis factor alpha, IL-1β: interleukin 1beta, IL-8: interleukin 8, HSP27: heat shock protein 27, MMP-2: matrix metalloproteinase-2, MMP-9: matrix metalloproteinase-2, RANKL: receptor activator of NF-κB ligand, OPG: osteoprotegerin, IL-17: interleukin 17, PTHrP: PTH-related peptide, miR-21: microRNA-21, miR-203: microRNA-203, ROS: reactive oxygen species, HIF-1α: hypoxia inducible factor 1 subunit alpha, PPARβ: peroxisome proliferator-activated receptor beta, HDACs: histone deacetylases, CD3+ cells: T cells, CD68+ cells: macrophages and monocytes, HSP60: heat shock protein 60, HSP70: heat shock protein 70.

**Table 4:** Roles of inflammatory mediators in triggering cholesteatoma lesions.

Inflammatory Molecules	Level of Evidence	Pathogenesis
IL-1alpha	More proof needed for its involvement	Could trigger epithelia cells hyperproliferation.
IL-6, IL-17	Not confirmed hypothesis	Could induce osteoclast proliferation
IL-8		Promotes local MMP production
EGF, TNF-α, TGFα	Both protective and destructive roles have been hypothesized	Modulate epithelial proliferation and angiogenesis
TLR4	Mediates the inflammatory response in acquired cholesteatoma	Possibly the promoters of ILs production; activate RANKL

IL-1alpha: interleukin 1 alpha, IL-6: interleukin 6, IL-17: interleukin 17, IL-8: interleukin 8, EGF: Epidermal growth factor, TNF-α: tumor necrosis factor alpha, TGFα: Tumor growth factor alpha, TLR4: Toll-like receptor 4, RANKL: receptor activator of NF-κB ligand.

**Table 5:** Roles of matrix metalloproteinases in cholesteatoma.

Type of MMP	Specimen Investigated	Pathogenesis
MMP-2	Tissues, blood	Could be involved in advanced/aggressive disease; not demonstrated in recurrent disease
MMP-8	Tissues	No role
MMP-9	Tissues, blood	Recurrence, when investigated in local tissues
MMP-13	Tissues	No role
MMP-14	Tissues	Possibly effects on bone destruction

MMP: Matrix metalloproteinase.

**Table 6:** Some miRNA possibly involved in cholesteatoma development.

MicroRNA Involved	Pathogenesis	Tissue Lesions Involved	Possible Interactions
miR-17	Upregulation of RANKL	Osteoclast differentiation	RANK/OPG
miR-21	Negatively regulates PTEN and PDCD4 (involved in cell growth inhibition and death)	Epithelial proliferation	STAT3 inhibitors
miR-802	Suppresses PTEN	Epithelial proliferation	NF-κB pathway
miR-142-5p	When suppressed, increases IL-6 and TNF-alpha production	Promotes inflammation	CDK5
miR-203a	Upregulates *Bmi1*	Promotes cell proliferation, inhibits apoptosis	p-Akt
miR-10a-5p	Upregulates *PIK3C*	Promotes cell growth, proliferation, and survival	PI3K-AKT signaling pathway
miR-34a and miR-125b	Inhibits apoptosis and proliferation	Promotes cell proliferation	STAT3 signaling

miR: microRNA, RANKL: Receptor activator of NF-κB ligand, RANK: Receptor activator of NF-κB, OPG: Osteoprotegerin, PDCD4: Programmed cell death protein 4, STAT3: Signal transducer and activator of transcription 3, PTEN: Phosphatase and TENsin homolog, NF-κB: Nuclear Factor kappa-light-chain-enhancer of activated B cells, IL-6: Interleukin 6, TNF-alpha: Tumor necrosis factor alpha, CDK5: Cyclin-dependent kinase 5, Bmi1: B lymphoma Mo-MLV insertion region 1 homolog, AKT: Protein kinase B, PI3K: Phosphatidylinositol 3-kinase.

## Abbreviations

The following abbreviations are used in this manuscript:

Akt	Protein kinase B
Bmi1	B lymphoma Mo-MLV insertion region 1 homolog
CD3+ cells	T cells
CD68+ cells	Macrophages and monocytes
CDK5	Cyclin-dependent kinase 5
CWU	Canal wall up technique
CWD	Canal wall down technique
ECE1	Endothelin converting enzyme 1
ECM	Extracellular matrix
EGF	Epidermal growth factor
FOXC2	Forkhead box C2
H3K27	Histone H3 at lysine 27
HDACs	Histone deacetylases
HIF-1α	Hypoxia inducible factor 1 subunit alpha
HSP27	Heat shock protein 27
HSP60	Heat shock protein 60
HSP70	Heat shock protein 70
IL-17	Interleukin 17
IL-1β	Interleukin 1beta
IL-6	Interleukin 6
IL-8	Interleukin 8
MBNL1	Muscleblind-like splicing regulator 1
miR-203	MicroRNA-203
miR-21	MicroRNA-21
miRNA	Micro RNA
MMP-2	Matrix metalloproteinase-2
MMP-9	Matrix metalloproteinase-9
NFAT5	Nuclear factor of activated T cells 5
NF-κB	Nuclear Factor kappa-light-chain-enhancer of activated B cells
OPG	Osteoprotegerin
PDCD4	Programmed cell death protein 4
PI3K	Phosphatidylinositol 3-kinase
PIK3CA	Phosphatidylinositol-4,5-bisphosphonate 3-kinase catalytic subunit α
PPARβ	Peroxisome proliferator-activated receptor beta
PTEN	Phosphatase and TENsin homolog
PTHrP	PTH-related peptide
RANK	Receptor activator of NF-κB
RANKL	Receptor activator of NF-κB ligand
ROS	Reactive oxygen species
SIRT	Sirtuin
STAT3	Signal transducer and activator of transcription-3
TGFBR2	Transforming growth factor beta receptor type 2
TGFα	Tumor growth factor alpha
TLR4	Toll-like receptor 4
TNF-α	Tumor necrosis factor alpha
